# Genome-wide copy number variant analysis reveals variants associated with 10 diverse production traits in Holstein cattle

**DOI:** 10.1186/s12864-018-4699-5

**Published:** 2018-05-02

**Authors:** Yang Zhou, Erin E. Connor, George R. Wiggans, Yongfang Lu, Robert J. Tempelman, Steven G. Schroeder, Hong Chen, George E. Liu

**Affiliations:** 1 Animal Genomics and Improvement Laboratory, BARC, USDA-ARS, 10300 Baltimore Avenue, Bldg. 306, BARC-East, Beltsville, MD 20705 USA; 2 Shaanxi Key Laboratory of Agricultural Molecular Biology, College of Animal Science and Technology, Northwest A&F University, Yangling, 712100 Shaanxi China; 3 Key Laboratory of Agricultural Animal Genetics, Breeding and Reproduction, Education Ministry of China, Huazhong Agricultural University, Wuhan, 430070 Hubei China; 4 Department of Animal Science, Michigan State University, East Lansing, MI 48824 USA

**Keywords:** Copy number variation (CNV), Dairy cow, Genome-wide association study, Feed intake, Feed efficiency

## Abstract

**Background:**

Copy number variation (CNV) is an important type of genetic variation contributing to phenotypic differences among mammals and may serve as an alternative molecular marker to single nucleotide polymorphism (SNP) for genome-wide association study (GWAS). Recently, GWAS analysis using CNV has been applied in livestock, although few studies have focused on Holstein cattle.

**Results:**

We describe 191 CNV detected using intensity data from over 700,000 SNP genotypes generated with the BovineHD Genotyping BeadChip (Illumina, San Diego, CA) in 528 Holstein cows. The CNV were used for GWAS analysis of 10 important production traits of 473 cattle related to feed intake, milk quality, and female fertility, as well as 2 composite traits of net merit and productive life. In total, we detected 57 CNV associated (*P* < 0.05 after false discovery rate correction) with at least one of the 10 phenotypes. Focusing on feed efficiency and intake-related phenotypes of residual feed intake and dry matter intake, we detected a single CNV associated with both traits which overlaps a predicted olfactory receptor gene *OR2A2* (*LOC787786*). Additionally, 2 CNV within the *RXFP4* (relaxin/insulin like family peptide receptor 4) and 2 additional olfactory receptor gene regions, respectively, were associated with residual feed intake. The *RXFP4* gene encodes a receptor for an orexigenic peptide, insulin-like peptide 5 produced by intestinal L cells, which is expressed by enteric neurons. Olfactory receptors are critical for transmitting the effects of odorants, contributing to the sense of smell, and have been implicated in participating in appetite regulation.

**Conclusions:**

Our results identify CNV for genomic evaluation in Holstein cattle, and provide candidate genes, such as *RXFP4*, contributing to variation in feed efficiency and feed intake-related traits. These results indicate potential novel targets for manipulating feed intake-related traits of livestock.

**Electronic supplementary material:**

The online version of this article (10.1186/s12864-018-4699-5) contains supplementary material, which is available to authorized users.

## Background

Apart from single nucleotide polymorphism (SNP), copy number variation (CNV) is another type of genetic marker that potentially affects animal phenotype [1]. These CNV consist of variation of genome sequences ranging from 50 to 5 million base pairs [2]. Compared to SNP, CNV show more drastic effects on gene expression and function, such as altering gene dosage, disrupting coding sequence, or perturbing long-range gene regulation [3]. However, until recently, the effective use of CNV as genetic markers for association with diseases and economic phenotypes has been impaired by difficulties in accurately detecting CNV and their boundaries, which vary among individuals [4, 5]. To date, especially within the livestock genomics field, most studies have focused exclusively on CNV discovery, and only a few studies and software have attempted to use comparative genomics methods to detect possible phenotype-related CNV in livestock using genome-wide association study (GWAS) [5–7].

Numerous methods have been used to detect CNV, ranging from traditional cytogenetic approaches, such as karyotyping and fluorescence in situ hybridization, to genome-wide in silico CNV prediction [8, 9]. Substantial improvements have been made recently to the accuracy and throughput of CNV discovery. For example, the application of microarray and next-generation sequencing technologies makes it possible to compare CNV among populations at a whole-genome scale, and sequencing methods offer opportunities to identify CNV in highly complex repetitive regions of the genome [10, 11]. However, neither sequencing methods are economical or widely used for large population analysis, including GWAS. Thus, the use of SNP chips is now commonplace for SNP-based GWAS studies, including studies of large populations of livestock. As a result, software packages have been developed and published for CNV prediction from SNP chip data, such as the SNP & Variation Suite (SVS; Golden Helix, Bozeman, MT), which facilitate a low-cost approach to detect common CNV within a population for GWAS analysis [5, 6].

Because Holsteins are the largest milk-producing dairy breed worldwide, GWAS examining reproduction, growth, milk production, and disease resistance traits among dairy cattle have been performed often in the Holstein breed. In these studies, SNP markers were used almost exclusively, whereas studies using CNV markers for GWAS are rare. For instance, Xu et al. [6] characterized 34 CNV significantly associated with milk production traits using a CNV inferred from the BovineSNP50 array (Illumina, San Diego, CA), and Durán Aguilar et al. [12] applied CNV-based GWAS for milk somatic score using the BovineHD Genotyping BeadChip (Illumina) containing over 777 K SNP [6, 12]. In a third study of Spanish Holsteins, 90 segregating CNV were identified from BovineSNP50 array hybridization signals and studied for their association with 7 production and conformation traits [7]. For other cattle breeds and livestock species, such as beef cattle and pigs, several GWAS have been conducted using CNV [5, 13].

Here, we applied CNV detection and GWAS analysis for 10 important production-related phenotypes of dairy cattle, including those related to feed intake, milk composition, female fertility, and productive life, as well as net merit. These studies were conducted using genotypes generated with the BovineHD Genotyping BeadChip (Illumina) from 528 Holstein cows. We identified 191 CNV based on size and frequency, and found 57 CNV associated with at least one of those phenotypes.

## Methods

### Animals

Cows (*n* = 528) used in the study were registered Holsteins from the Beltsville Agricultural Research Center Dairy Herd located in Beltsville, MD. Genotyping was performed using high-quality genomic DNA extracted from white blood cells or hair follicles of cows using Gentra Puregene DNA extraction kits (Qiagen, Valencia, CA) and the BovineHD Genotyping BeadChip (Illumina).

### Phenotypes and PTA calculations

Phenotypes were available for analysis from 473 of the genotyped cows. Measurements for estimation of DMI and RFI were performed as described previously [14]. Production traits of cows were collected by Dairy One Cooperative Inc. (Ithaca, NY) using ICAR-approved methods and quality certification standards administered by the Council on Dairy Cattle Breeding (Bowie, MD). Conventional PTA for RFI and DMI were calculated as described in Lu et al. [15], and PTA for milk fat percentage, milk protein percentage, cow conception rate, heifer conception rate, daughter pregnancy rate, somatic cell score, net merit, and productive life were calculated as described in VanRaden and Wiggans [16]. De-regressed PTA (dPTA) were calculated according to the formula: dPTA = PTA / reliability as described by Garrick et al. [17] and used as the phenotype for further CNV GWAS, similar to Xu et al. [6]. Although matrix deregression based on pedigree structure is expected to more accurately remove the contributions of other relatives to the final deregressed evaluation [18], we used the simpler removal procedure.

### CNV segmentation and genotyping

The SNP genotypes generated by the Illumina BovineHD Genotyping BeadChip assay with a call rate > 90% were used to detect the common CNV shared among Holstein cows using methods previously described by Zhou et al. [5]. Specifically, the DSF file was exported from GenomeStudio Software and the Log R Ratios (LRR) were imported into SVS 8.3.0 ([19]; Golden Helix Inc.). A total of 735,293 SNP were successfully mapped onto the 29 autosomes of the *Bos taurus* genome assembly UMD 3.1 [20]. The default GC correction file in the SVS software was used to correct the LRR waviness caused by the GC content. The multivariate method was used to define the CNV segments, employing the SVS default settings which are based on the expected CNV density (count and length) within a genome, as follows: (1) a maximum of 20 segments per 10 K markers; (2) at least 3 markers per segment; and (3) a maximum pair-wise segment *P*-value of 0.005. The 3 states with a comparatively strict threshold (segment mean ± 0.4) defined the CNV as 3 types of events (loss [− 1], neutral [0], or gain [+ 1]) across all of the samples.

### PCA-corrected association testing

Multiple linear regression in an additive genetic model was used to identify the CNV significantly associated with 10 traits individually. Both FDR and 10,000 permutations were performed to correct for multiple testing. The model was as follows:

$$ {y}_i=\sum \limits_{j=1}^m{x}_{ij}{\beta}_j+{e}_i $$

where *y*_*i*_ is the dPTA of the *i*^th^ individual, *x*_*ij*_ is the CNV genotype of the *i*^th^ individual (gain, loss, and neutral as represented by 1, − 1 and 0), *β*_*j*_ is the CNV effect, *m* is the number of CNV, and *e*_*i*_ is the residual. To correct for batch effects/stratification, the Principal Component Analysis (PCA) option was selected, wherein the SVS software identified the optimum number of components based on a range from 1 to 20. See Golden Helix SNP & Variation Suite v8.3.0 Manual [19] for additional details on methodology. Significant CNV were identified after false discovery rate (FDR) correction (*P* < 0.05) [6].

### Evaluation of QTL and genetic overlap of CNV

Gene annotations were downloaded from the Ensembl database [21] and QTL were downloaded from the animal QTL database [22] (based on the UMD3.1 bovine reference genome assembly). The overlaps between CNV and genes or QTL were detected using R3.3.3 GenomicRanges [23]. To detect potential genes affected by significant CNV, we define the ‘overlap’ as more than 1 bp in common between the CNV region and the genomic region (including the 5-Kb flanking regions both up- and downstream) of a given gene transcript. Because of overly large confidence intervals (reported by the Animal QTLdb) for some QTL, we filtered out the QTL with confidence intervals > 30 Mb and used a strict threshold to define the overlap as at least 50% of the CNV length being covered by the QTL.

### CNV validation by qPCR

For each CNV selected for validation, the Illumina Bovine HD SNP chip probe sequences corresponding to SNP within the CNV region of interest were identified. The 400-bp genomic DNA sequence surrounding each targeted SNP (i.e., ± 200 bp of the SNP) was selected as the template sequence for PCR primer design using the NCBI Primer-BLAST primer designing tool [24]. The desired PCR product size was specified as 100 to 300 bp and the best primer pair was selected from the output. Primer information can be found in Additional file 2: Table S1. Reactions (25 μL) were performed in triplicate using SsoAdvanced Universal SYBR Green Supermix (Bio-Rad, Hercules, CA), 5 ng genomic DNA, and 400 n*M* each primer on the Bio-Rad CFX96 Touch Real-Time PCR Detection System. Amplification consisted of 95 °C for 1 min, followed by 45 cycles of 95 °C for 10 s, 58.2 °C for 10 s, and 72 °C for 30s. Amplification of the expected product size was confirmed by electrophoresis on a 2% agarose gel. Melting curve analysis following the final PCR amplification step was used to confirm presence of a single amplicon. Efficiency of PCR amplification for each target was determined from a 4-point standard curve using 1.5 to 24 ng of genomic DNA as template, and ranged from 99.3 to 107.4%. Linearity of all standard curves exceeded 0.995. The *BTF3* gene and a common DNA sample from Hereford cow, L1 Dominette 01449 were used as references for all qPCR experiments. The 2^-ΔΔC^_T_ method [25] was employed to analyze qPCR results wherein we defined 0 to 1.5 copies as a copy loss (− 1), > 1.5 to 3 copies as neutral (0), and > 3 copies as gain (+ 1).

### Phenotype correlation analysis

Ten diverse phenotypes of RFI, DMI, milk fat percentage, milk protein percentage, somatic cell score, cow conception rate, heifer conception rate, daughter pregnancy rate, net merit, and productive life for 473 Holstein cows were selected for correlation analysis to understand their relationships to one another. Pearson product-moment correlations were computed between pairs of dPTA for all of the 10 phenotypes.

## Results and discussion

### CNV segments and genotyping

**Fig. 1:**
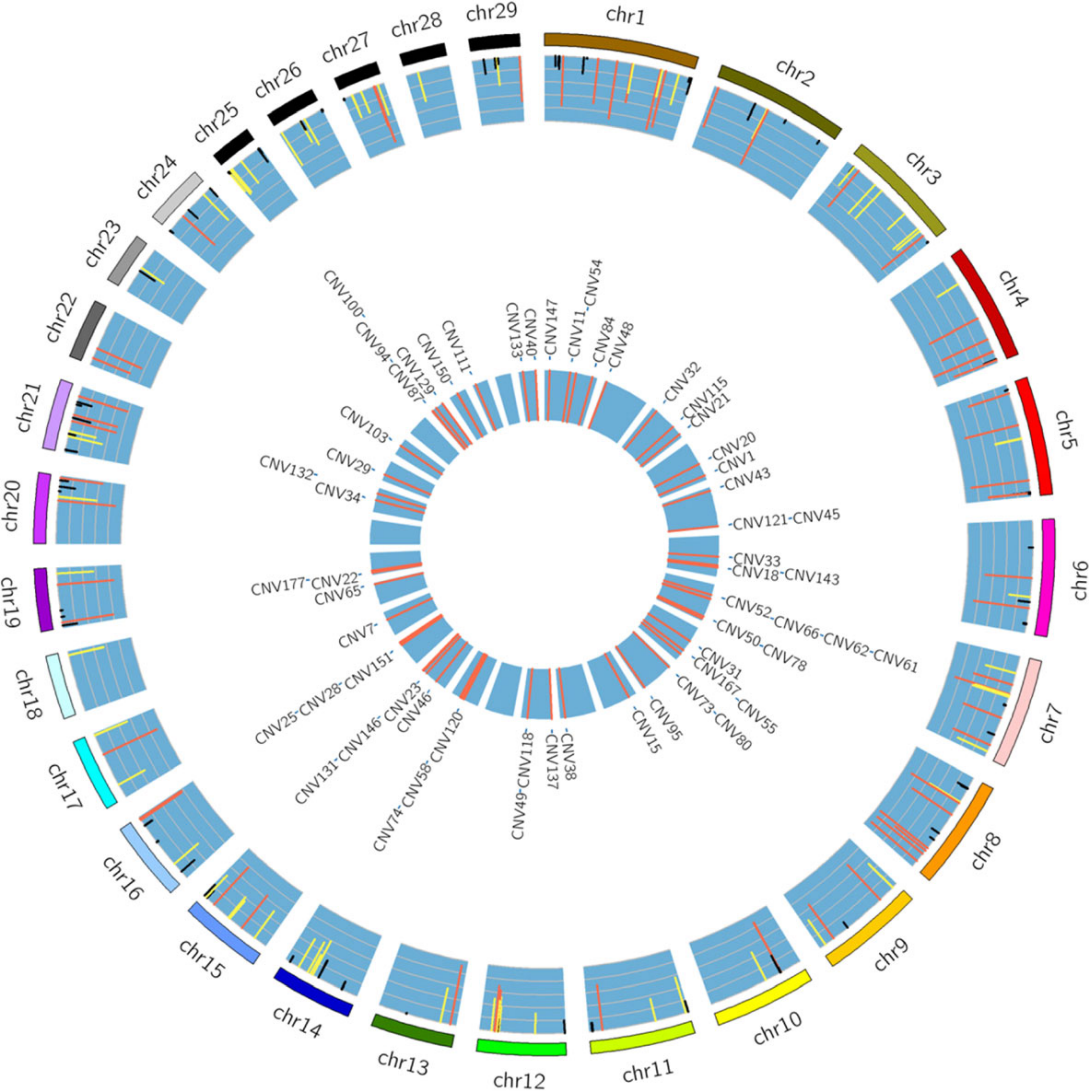
Distribution of CNV and significantly associated CNV on each *Bos taurus* autosome. Outer circle: distribution of 191 CNV where the height and color of histograms represent the variant frequency of each CNV (red, > 0.6; yellow, 0.3 to 0.6; and black, < 0.3); Inner circle: distribution of CNV significantly associated with at least 1 of the 10 production traits evaluated

We detected 454 CNV from 528 Holstein cows where the CNV were characterized as 3 types (− 1, 0, and + 1). We filtered out likely false CNV using the strict threshold criteria of length ≤ 1 Mb and frequency > 0.5%. The resulting 191 common CNV had lengths ranging from 727 to 897,251 bp and frequencies ranging from 1.1 to 98.9% (Additional file 2: Table S2; Fig. 1). These CNV comprised 0.64% (16.2 Mb) of the whole autosomal length and were distributed differently among autosomes. The chromosome with the highest percentage of CNV length (3.8%) was Chr15. Bovine Chr12 was reported as having the highest percentage of CNV length in a previous CNV study [11], whereas in the current study, Chr12 ranked second highest for percentage of CNV length among all autosomes. We detected 164 genes that overlapped with the CNV (Additional file 2: Table S2). A total of 47 or 189 of our 191 CNV events were overlapped with Ben Sassi et al. and Xu et al. (Additional file 2: Table S2) [6, 7]. Gene ontology (GO) analysis results of genes overlapping with CNV are typically enriched in functions related to immunity or response to stimuli [26]. However, in our DAVID analysis using Fisher’s exact test, the CNV were highly enriched in GO terms related to development and growth, such as: multicellular organismal process, developmental process, and multicellular organismal development (Additional file 2: Table S3).

### Phenotype correlation analysis

**Fig. 2:**
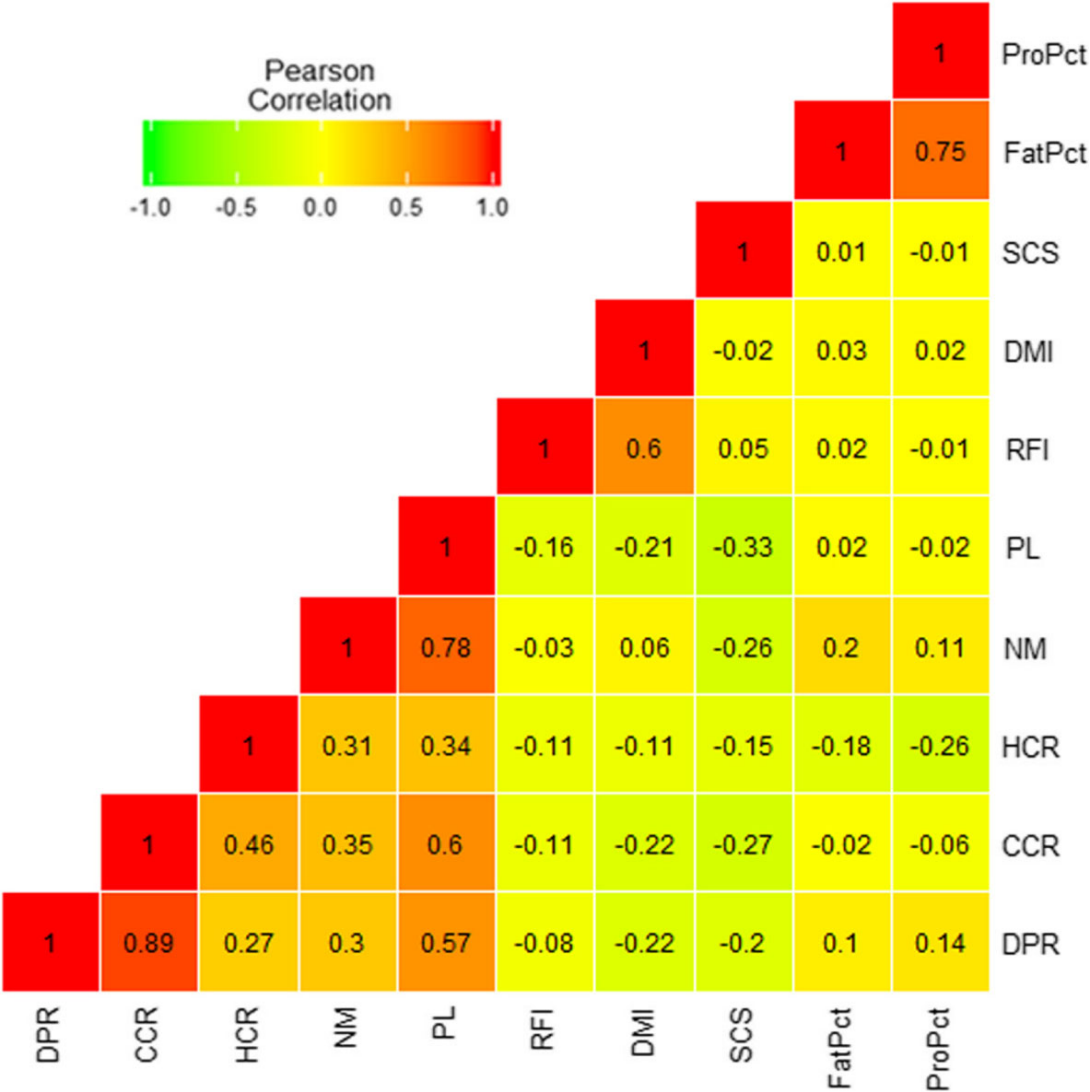
Pair-wise Pearson correlation coefficients for 10 dairy production-related phenotypes of interest. DPR: daughter pregnancy rate; CCR: cow conception rate; HCR: heifer conception rate; NM: net merit; PL: productive life; RFI: residual feed intake; DMI: dry matter intake; SCS: somatic cell score; FatPct: fat percentage; and ProPct: protein percentage

Figure 2 shows results of the Pearson correlation analysis between each of the dPTA. As described previously [27, 28], the 10 dPTA generally showed moderate to strong correlation coefficients within each phenotypic category (i.e., feed efficiency and intake [RFI and DMI], milk composition and quality [fat percentage, protein percentage, and somatic cell score], fertility [cow conception rate, heifer conception rate, and daughter pregnancy rate], and composite evaluation [net merit and productive life]), but weaker correlation coefficients between different phenotypic categories. For example, RFI versus DMI was moderate (*r* = 0.60, *P* < 0.0001), milk fat versus protein percentage was moderate to high (*r* = 0.75, *P* < 0.0001), and the correlation coefficients of the 3 fertility traits were variable, ranging from 0.27 to 0.89. Net merit and productive life were highly correlated (*r* = 0.78, *P* < 0.0001) with one another, and both were moderately correlated with the fertility phenotypes. Somatic cell score showed weak correlation with other phenotypes, such as productive life (*r* = − 0.33, *P* < 0.0001) and net merit (*r* = − 0.26, *P* < 0.0001), and most coefficients were negative.

### Significant CNV association analysis

**Fig. 3:**
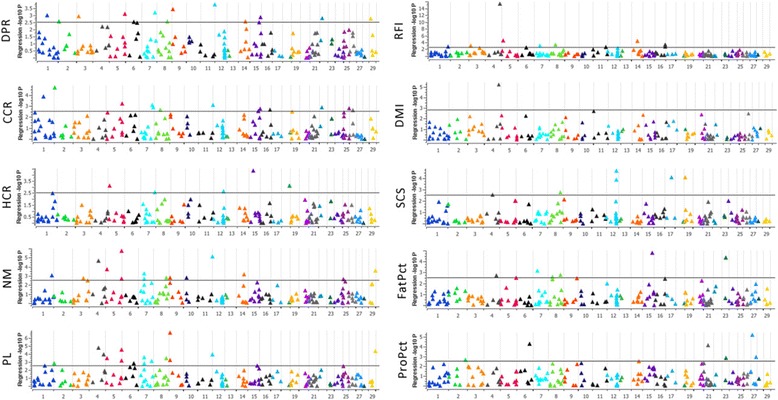
Manhattan plots of the genome-wide association study results for 10 dairy production traits of interest. Negative log_10_-transformed *P*-values from a genome-wide scan (y-axis) are plotted against genomic coordinates on 29 *Bos taurus* autosomal chromosomes (x-axis). The solid horizontal line in each plot represents the threshold for significance based on a *P*-value < 0.05 after FDR correction. DPR: daughter pregnancy rate; CCR: cow conception rate; HCR: heifer conception rate; NM: net merit; PL: productive life; RFI: residual feed intake; DMI: dry matter intake; SCS: somatic cell score; FatPct: fat percentage; and ProPct: protein percentage

Before applying the GWAS analysis, we excluded 2 CNV because they appeared in fewer than 5 individuals. The frequency of the remaining 189 CNV among the 473 cows ranged from 1.3 to 98.9%. In total, we detected 57 CNV associated after FDR correction (*P* < 0.05) with at least 1 of the 10 phenotypes (Additional file 2: Table S4; Fig. 3). These significant CNV were distributed among 25 autosomal chromosomes (Fig. 1), wherein no significant CNV were identified on Chr13, Chr20, Chr24, or Chr28. We did not observe strong bias due to the frequency of CNV present on a given chromosome on the number of significant CNV detected within that chromosome. Chr1 and Chr12 had the greatest CNV number for GWAS analysis, but based on CNV density (i.e., CNV count normalized by chromosome length), we did not find obvious enrichment of significant CNV on these 2 chromosomes. However, Chr7 was enriched with the greatest number (*n* = 6) of CNV significantly associated with the production traits of interest. The length of the 57 significant CNV ranged from 1.2 to 350 Kb and their frequencies ranged from 4.6 to 98.9%. We selected 7 significant CNV associated with multiple traits of interest or overlapping known genes or QTL for validation among 8 animals using qPCR. As most of the CNV (90%) were deletions, all 7 selected CNV were deletions. For 3 out of 7 cases, the PCR amplicon for CNV validation was within genes: CNV33 (*RHOH*), CNV46 (*GRIK4*), and CNV40 (*AP2A2*). The result showed 55.4% of the 56 qPCR results were consistent with the in silico prediction (Additional file 2: Table S5), which is typically in the range of 60 to 70% concordance [29]. Inconsistencies between qPCR and in silico prediction may occur due to multiple reasons, such as complex sequence of the genome, artificial assembly, probe bias, primer design, DNA quality, or other factors during array hybridization or PCR amplification.

As expected, the significant CNV were associated with phenotypes of different categories, with the number of significant CNV associated with a given trait ranging from 1 (e.g., dry matter intake) to 19 (e.g., productive life; Additional file 2: Table S4). Phenotypes with higher correlation are more likely to share significant CNV. For example, we found 6 significant CNV (CNV11, CNV23, CNV29, CNV50, CNV121, CNV137) associated with both cow conception rate and daughter pregnancy rate; traits which were highly correlated. Similarly, another highly correlated phenotypic pair of net merit and productive life shared 10 significant CNV (CNV20, CNV40, CNV43, CNV45, CNV55, CNV62, CNV80, CNV94, CNV118, CNV121), whereas RFI and DMI shared only 1 significant CNV (CNV1). The most dissimilar phenotype in the correlation analysis, somatic cell score, was found to share no significant CNV with any of the other phenotypes evaluated. The relationships between phenotypes will help to better define potentially related markers as significant CNV appearing with both or all highly correlated phenotypes will be more reliable than those CNV associated with only 1 phenotype.

### Genes and QTL overlapping with significant CNV

We detected 54 Ensembl gene ID overlapping with 20 significant CNV (Additional file 2: Table S4). Among them, CNV150 (Chr26: 25,719,640–26,013,587) overlapped with the greatest number of genes (*n* = 13) with all genes located inside the CNV region. There were 11 significant CNV (CNV147, CNV32, CNV33, CNV18, CNV66, CNV146, CNV151, CNV177, CNV34, CNV150, and CNV40) that overlapped with genes previously reported in cattle, or other species, to harbor deletions or duplications, and several others overlapping known gene regions. For instance, *ELF3* was identified as a candidate gene for somatic cell score in a previous CNV-based GWAS study [12]. In the present study, CNV151 was associated with cow conception rate and is overlapping with the *ELF3* gene. The product of the *ELF3* gene is a transcription factor that functions in inflammation and epithelial cell differentiation, and may be involved in mammary gland development and involution [30], supporting its potential link to somatic cell score. How its function may relate to cow conception rate is not known.

Over half of the significant CNV were overlapping with at least 1 QTL (Additional file 2: Table S4), of which some were for traits that support our GWAS results. For example, CNV46 was significantly associated with heifer conception rate and it overlaps with a QTL for the circulating level of the reproductive hormone, LH. In addition, CNV23 was significantly associated with cow conception rate and daughter pregnancy rate, and it overlaps with a QTL for first-service conception rate. Lastly, CNV66 was significantly associated with RFI and overlaps with a QTL for average daily weight gain, and CNV7 was associated with somatic cell score and overlaps with multiple QTL for somatic cell score and clinical mastitis. Thus, further evaluation of these CNV is warranted in additional cattle populations for their association with these, or similar, production traits.

### Characterization of shared significant CNV for phenotypes related to feed efficiency and intake

We focused our study on the 2 phenotypes of RFI and DMI related to feed efficiency and intake. There were 10 and 1 CNV significantly associated with RFI and DMI, respectively (Table 1). The CNV shared by RFI and DMI was CNV1 (Chr4: 108,225,979–108,252,635; *P* ≤ 0.001), which overlaps predicted olfactory receptor gene *OR2A2* (*LOC787786*). However, only 6 cows were typed for CNV1 as neutral (0), while the remaining 467 cows were all typed as loss (− 1). Although the 6 neutral cows had a greater mean dPTA for both RFI and DMI, we identified some cows possessing the CNV1 loss type that had equal or even higher dPTA than the mean dPTA of the CNV1 neutral types (Additional file 1: Figure S1). Thus, particular attention must be given to significant CNV occurring at very low or high frequency when considering them as candidate markers for use in animal breeding.

**Table 1:** Significant CNV from genome-wide association analysis for RFI and DMI traits among Holstein cattle

				*P*-value after FDR correction		Overlapped Ensemble		
CNV	Chromosome	Start (bp)	End (bp)	DMI	RFI	Gene ID	Gene Symbol	Overlapped QTL
CNV1	4	108,225,979	108,252,635	**0.0012**	**0.0000**		*LOC787786* (*OR2A2*)	
CNV25	16	79,851,676	79,853,884	0.2802	**0.0236**			
CNV28	16	78,028,784	78,035,505	0.9670	**0.0367**	ENSBTAG00000002291	*ZBTB41*	
CNV31	8	44,934,828	44,944,671	0.3442	**0.0201**		*LOC101909075*	
CNV32	3	14,876,353	14,882,132	0.1561	**0.0259**	ENSBTAG00000046885	*RXFP4*	
CNV38	11	93,188,055	93,190,178	0.6321	**0.0391**			Stearic acid content
CNV43	5	9,756,491	9,757,695	0.4402	**0.0027**			Infectious bovine keratoconjunctivitis susceptibility, Cold tolerance, Calving ease (maternal), Male fertility, Gestation length, Milk alpha-lactalbumin %, Retained placenta (DYD), Milk protein %
CNV66	7	42,745,346	42,788,788	0.4395	**0.0274**	ENSBTAG00000046318	*LOC787816* (*OR2T12*)	Cold tolerance
						ENSBTAG00000007557	*OR2AK2*	Average daily gain
CNV84	1	143,922,352	143,945,292	0.7750	**0.0357**			
CNV120	14	44,862,013	44,868,983	0.3398	**0.0025**			Subcutaneous fat

Note: See Additional file 2: Table S4 for additional details. *P*-values < 0.05 are indicated in bold font

The CNV32 (Chr3: 14,876,353–14,882,132) associated with RFI also only showed loss and neutral types in our population, with a loss frequency of 81.0%. It is located 3.7 Kb upstream of the *relaxin/insulin like family peptide receptor 4* (*RXFP4*; Chr3:14,871,054–14,872,623;strand: -) gene transcription start site, which is potentially affecting *RXFP4* gene expression by altering its promoter region. Of interest, the ligand for RXFP4 is insulin-like peptide 5 (Insl5), which is produced by intestinal L cells in response to a reduction in feed intake, stimulating appetite [31]. In humans, *RXFP4* was found to be significantly associated with obesity and body mass index [32]. Studies also reported that Insl5-RXFP4 signaling plays a role in glucose metabolism [33]. Thus, CNV32 may affect feed efficiency of cattle through an *RXFP4*-mediated pathway and provides an interesting candidate gene for further study in cattle.

Another significant CNV associated with RFI, CNV66 (Chr7:42,745,346–42,788,788), showed both loss and gain types with a total frequency of 54.6% within our Holstein population. Similar to CNV1, 2 olfactory receptor genes of predicted *OR2T12* (*LOC787816*) and *OR2AK2* are located inside CNV66. Olfactory receptors may influence feeding behavior, such as food preference and feed intake [34]. The variation of olfactory receptor gene copy numbers, theoretically, could affect their expression levels and impact RFI and DMI of Holstein cattle. These olfactory receptor genes serve as additional candidates for further study in the regulation of feed efficiency and feed intake of dairy cattle.

## Conclusions

Our previous study showed that ~ 25% of CNV did not have a significant association with SNP; thus the effects of these CNV probably were not captured by tag SNP [6]. In this study, we performed a CNV-based GWAS for 10 important production traits and detected 57 CNV significantly associated with at least one of these production traits. Of particular interest regarding feed intake-related phenotypes, we detected 2 CNV associated with RFI located within *RXFP4*, encoding a G-protein coupled receptor thought to play a role in regulation of appetite and metabolism [31, 35], and 2 olfactory receptor gene regions, respectively. We also identified a single CNV within predicted *OR2A2* strongly associated with both RFI and DMI. Our results identify CNV for genomic evaluation in Holstein cattle, and provide candidate genes contributing to variation in feed efficiency and feed intake-related traits.

## Additional files

Additional file 1: **Figure S1.** Box plot for dPTA distribution of the 2 feed intake-related phenotypes of RFI and DMI. (PDF 90 kb)

Additional file 2: **Table S1.** Primer information for CNV validation. **Table S2.** Characteristics of CNV and their overlapped genes. **Table S3.** Gene ontology (GO) of genes that overlap with CNV. **Table S4.** CNV-based GWAS results for the 10 phenotypes of interest among Holstein cattle. **Table S5.** qPCR validation of the significant CNV. (XLSX 96 kb)
