# Supplementary material for: Genome-wide copy number variant analysis reveals variants associated with 10 diverse production traits in Holstein cattle
Source: BMC Genomics. 2018 May 2;19:314. doi: 10.1186/s12864-018-4699-5 (PMC5930521; doi:10.1186/s12864-018-4699-5)
Supplement: Supplementary file 1 — Figure S1. Box plot for dPTA distribution of the 2 feed intake-related phenotypes of RFI and DMI. (PDF 90 kb) [file 12864_2018_4699_MOESM1_ESM.pdf]

Fig. S1 Box plot for dPTA distribution of the 2 feed intake-related phenotypes of RFI and DMI. CNV type: -1 = loss (n = 467 cows); 0 = neutral (n = 6 cows). Only 2 CNV genotypes had significant associations with RFI and DMI.

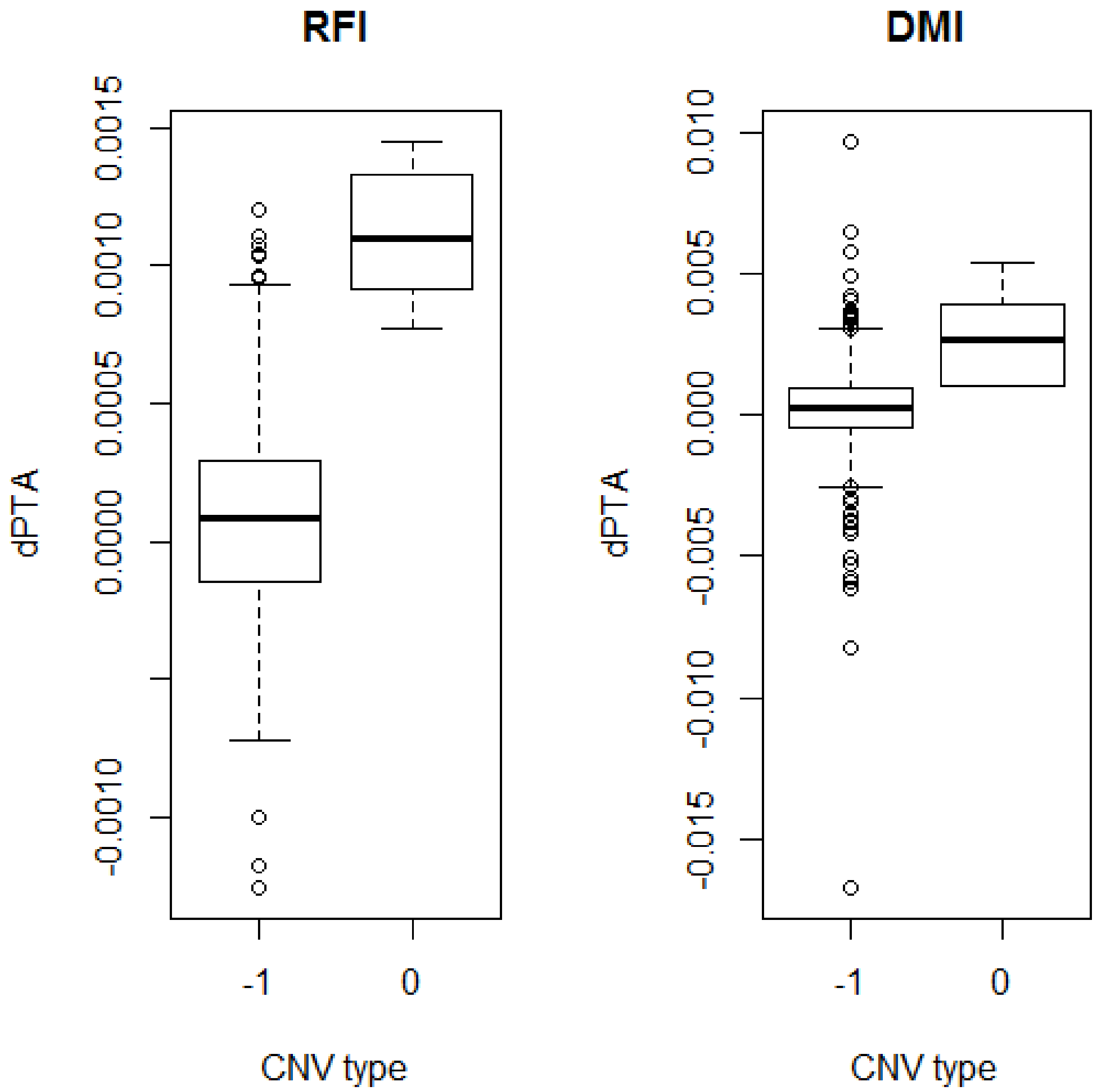
RFI
dPTA
0.0015
0.0010
0.0005
0.0000
-0.0010
-1
0
CNV type
DMI
dPTA
0.010
0.005
0.000
-0.005
-0.010
-0.015
-1
0
CNV type
